# Recall of reactive cutaneous capillary endothelial proliferation (RCCEP) induced by stereotactic body radiation therapy (SBRT) in a patient with lung squamous cell carcinoma

**DOI:** 10.1186/s40164-022-00336-4

**Published:** 2022-10-26

**Authors:** Rong Ma, Jia-Lin Wang, Yan-Yang Wang

**Affiliations:** 1 Department of Radiation Oncology, General Hospital of Ningxia Medical University, Ningxia 750004 Yinchuan, China; 2 Cancer Institute, Ningxia Medical University, Ningxia 750004 Yinchuan, China

**Keywords:** Lung cancer, Reactive cutaneous capillary endothelial proliferation (RCCEP), Stereotactic body radiation therapy (SBRT), Recall

## Abstract

Camrelizumab (SHR-1210) is a humanized IgG4 monoclonal anti-programmed cell death protein 1 (PD-1) antibody that has been shown to inhibit the binding of PD-1 to PD-L1, thereby blocking the immune escape of various types of cancer, including lung squamous cell carcinoma (LSCC). Reactive cutaneous capillary endothelial proliferation (RCCEP) is the most common adverse event in camrelizumab-treated patients. Here, we introduce a case of LSCC with recall RCCEP induced by stereotactic body radiation therapy (SBRT). A 76-year-old LSCC patient developed RCCEP when he received camrelizumab and chemotherapy. After discontinuing camrelizumab treatment, the RCCEP lesions spontaneously regressed and fell off. However, when the patient received subsequent SBRT, the RCCEP occurred again at the same sites. This case may provide clues for additional study of the immune reactivation effect of SBRT or the underlying mechanism of RCCEP.

**Dear Editor**

Immune checkpoint inhibitors (ICIs) have revolutionized the treatment of lung cancer. Camrelizumab is an antibody directed against programmed death protein 1 (PD-1) developed by Jiangsu Hengrui Pharmaceutical Co., Ltd. It blocks the immune escape of various types of cancer, including lung squamous cell carcinoma (LSCC). In the CameL-sq study, a phase III trial evaluating the efficacy and safety of camrelizumab combined with chemotherapy as a first-line treatment for advanced LSCC, compared with placebo, camrelizumab significantly prolonged the progression-free survival (PFS) and overall survival (OS) of LSCC patients [1].

Reactive cutaneous capillary endothelial proliferation (RCCEP) is the most common adverse event observed in camrelizumab-treated patients [2]. In the CameL-sq study, the incidence of RCCEP in patients with LSCC who received camrelizumab plus chemotherapy was 69%. Here, we introduce a case of LSCC with recall RCCEP induced by stereotactic body radiation therapy (SBRT). An LSCC patient developed RCCEP when he received camrelizumab and chemotherapy. After discontinuing camrelizumab treatment, his RCCEP lesions spontaneously regressed and fell off. However, when the patient received subsequent SBRT, the RCCEP occurred again at the same sites.

In March 2021, a 76-year-old man was diagnosed with T2N0M0 LSCC according to the eighth edition of the AJCC TNM Staging System. Due to his poor lung function, the patient first received camrelizumab (200 mg, every 3 weeks) plus paclitaxel (240 mg, every 3 weeks) and carboplatin (500 mg, every 3 weeks) and achieved a partial response (PR). In May 2021, the treatment was switched to tislelizumab (200 mg, every 3 weeks) plus paclitaxel (240 mg, every 3 weeks) and carboplatin (500 mg, every 3 weeks) due to grade 3 “red-nevus-like” and “pearl-like” RCCEP induced by camrelizumab. The RCCEP lesions regressed within 1 month after the withdrawal of camrelizumab treatment. The diagnosis of RCCEP was made based on the context and a physical examination conducted by a physician.

In September 2021, after four cycles of tislelizumab plus chemotherapy, the anticancer treatment of the patient was interrupted by COVID-19. No grade 3 or higher immune-related adverse events (irAEs) were observed during tislelizumab treatment. In November 2021, a chest CT scan showed a significant increase in the size of the primary lung lesion, indicating the progression of the disease. The patient was then referred for SBRT treatment. The primary lung lesion was irradiated with a total dose of 48 Gy in 8 fractions.

Nevertheless, 2 weeks after the start of SBRT, the patient developed dyspnea, dry cough, and low-grade fever. A chest CT scan showed changes suggesting pneumonia, especially in the area of the left lung that was previously exposed to radiation (Fig. 1). Bacterial or fungal infection was ruled out, and the patient was diagnosed with radiation-induced pneumonia. At the same time, grade 2 “red-nevus-like” RCCEP appeared on the patient’s trunk (Fig. 2). Corticosteroids were immediately started and then tapered for more than 6 weeks. The symptoms of radiation-induced pneumonia were relieved rapidly after treatment. In January 2022, a repeated chest CT scan revealed that the radiation-induced pneumonia was significantly improved. The recall RECCP fell off 2 months after starting corticosteroid treatment.

**Fig. 1:**
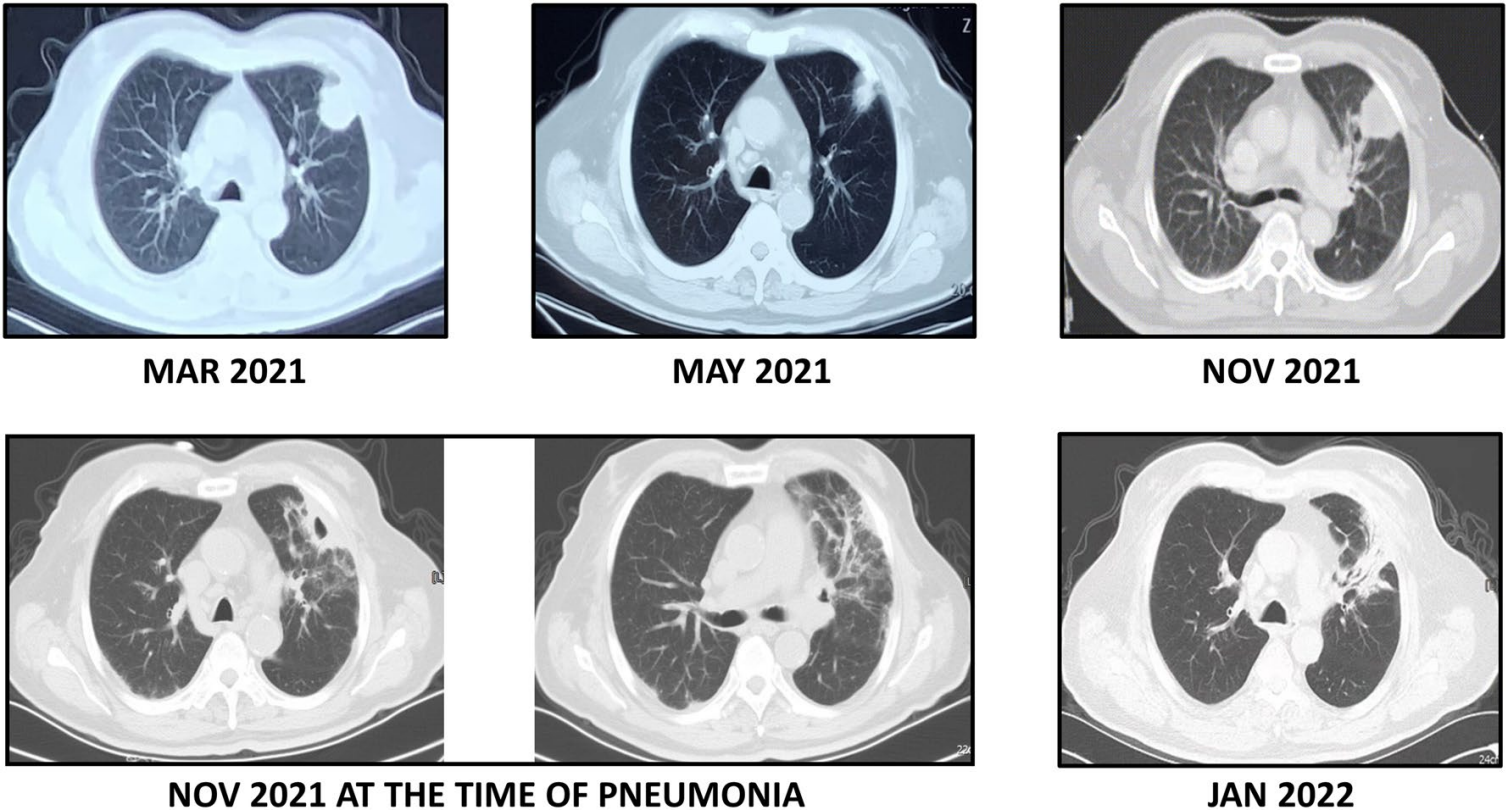
Chest CT scans of the patient with lung squamous cell carcinoma (LSCC) at different time points during treatment

**Fig. 2:**
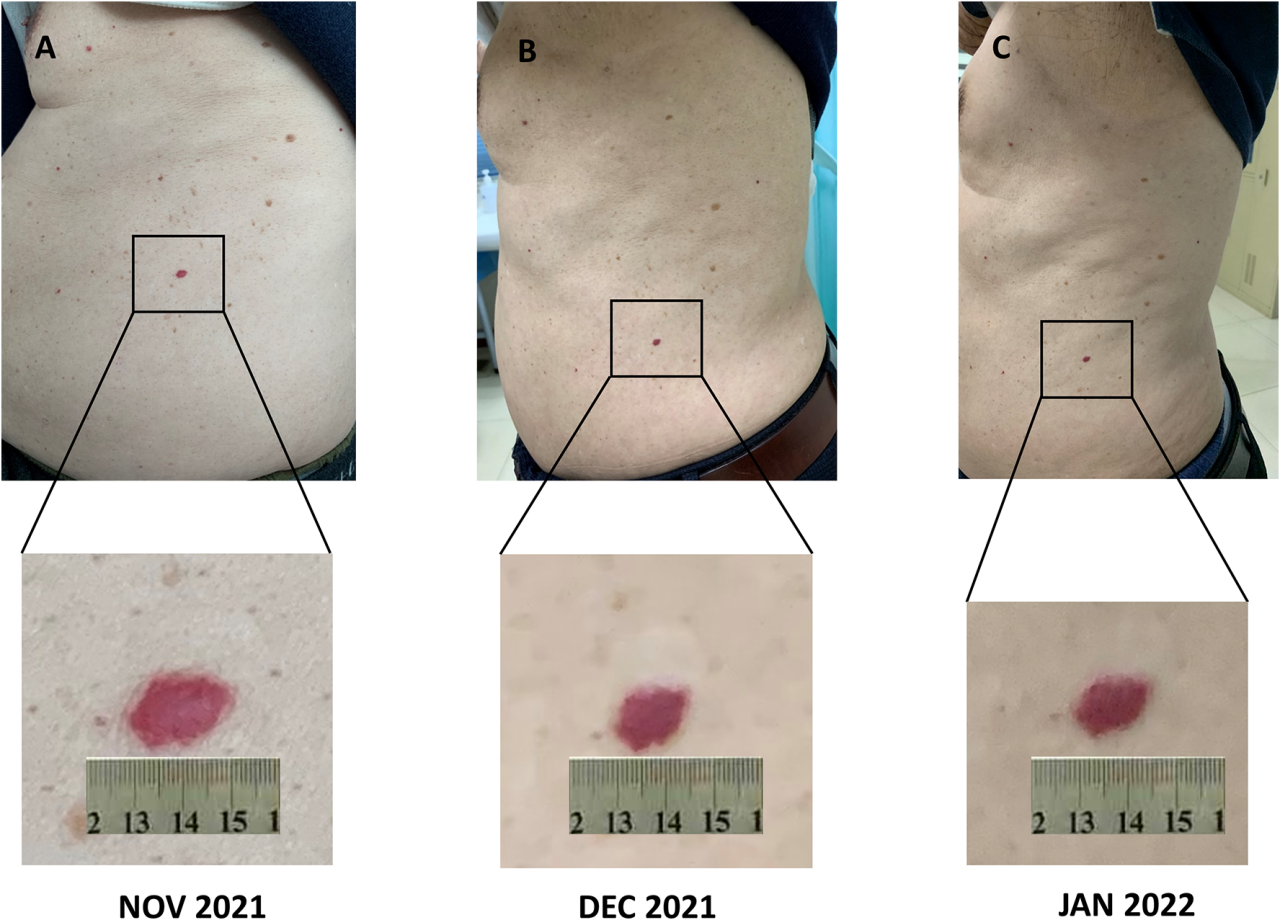
The recall reactive cutaneous capillary endothelial proliferation (RECCP) lesion caused by stereotactic body radiation therapy (SBRT)

RCCEP is a common irAE of camrelizumab that occurs mainly on the skin of the head, face, trunk, and extremities. Most RCCEP cases occur 2–4 weeks after starting camrelizumab treatment. According to the changes in the lesions, RCCEP can be divided into 5 types: “red-nevus type”, “pearl-type”, “mulberry-type”, “patch-type”, and “tumor-type”. Although most RCCEPs are grade 1 or 2, occasional cases of severe grade 3 to 4 RCCEPs can lead to discontinuation of treatment and life-threatening complications [3]. The exact mechanism of RCCEP development is not clear and may be related to the imbalance between pro- and antiangiogenic factors caused by immune reactivation. Vascular endothelial growth factor-A (VEGF-A) and VEGFR2-pY1175 are highly expressed in RCCEP lesions. Therefore, the activation of VEGFR2 mediated by the immune response may be involved in the pathogenesis of RCCEP [4].

Management guidelines for RCCEP have not yet been developed. Although RCCEP is a kind of irAE, it is not sensitive to glucocorticoids. In fact, most RCCEP lesions are self-limiting and do not require intervention. Local treatment, such as laser therapy, minor resection, hemostatic therapy, local hormone therapy, cryotherapy, etc., can be applied in special circumstances [4]. In addition, camrelizumab combined with chemotherapy agents or apatinib can significantly reduce the incidence of RCCEP [1, 5]. In some studies, RCCEP has also been shown to be a clinical biomarker for predicting the anti-cancer efficacy of camrelizumab [4].

Re-occurrence of RCCEP at previous sites has been observed when restarting camrelizumab [6]. However, the regrowth of RCCEP triggered by other treatment modalities has not been reported previously. In this case, we found that SBRT could induce the recall of RCCEP. Camrelizumab is becoming an increasingly popular ICI in cancer treatment. It is important to pay attention to the recall of RCCEP. At the same time, this case also provides clues for further study of the immune reactivation effect of SBRT and the mechanism underlying the occurrence of RCCEP.

## Data Availability

The data used and/or analyzed during the current study are available from the corresponding author on reasonable request.
